# Supplementary material for: Human Serum Proteins and Susceptibility of Acinetobacter baumannii to Cefiderocol: Role of Iron Transport
Source: Biomedicines. 2022 Mar 3;10(3):600. doi: 10.3390/biomedicines10030600 (PMC8945497; doi:10.3390/biomedicines10030600)
Supplement: Supplementary file 1 [file biomedicines-10-00600-s001.zip › biomedicines-1620747-supplementary.pdf]

**A**

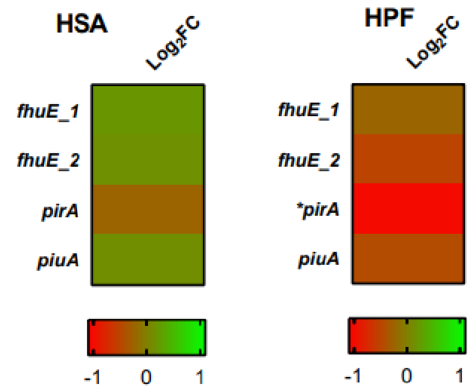


| gene | | | HSA | | HPF | |
|---|---|---|---|---|---|---|
| | | | $log_2$ fold change | adjusted *p-value* | $log_2$ fold change | adjusted *p-value* |
| A591_A1573 | ABUW_1655 | *fhuE_1* | 0,191704 | 0,784416 | -0,231239 | 0,6952062 |
| A591_A2128 | ABUW_2165 | *fhuE_2* | 0,135697 | 0,676467 | -0,515881 | 0,0033339 |
| A591_A3336 | ABUW_3403 | *pirA* | -0,23853 | 0,350777 | -1,017631 | 2,619E-11 |
| A591_A2869 | ABUW_2916 | *piuA* | 0,121735 | 0,747457 | -0,452773 | 0,0318274 |

**B**

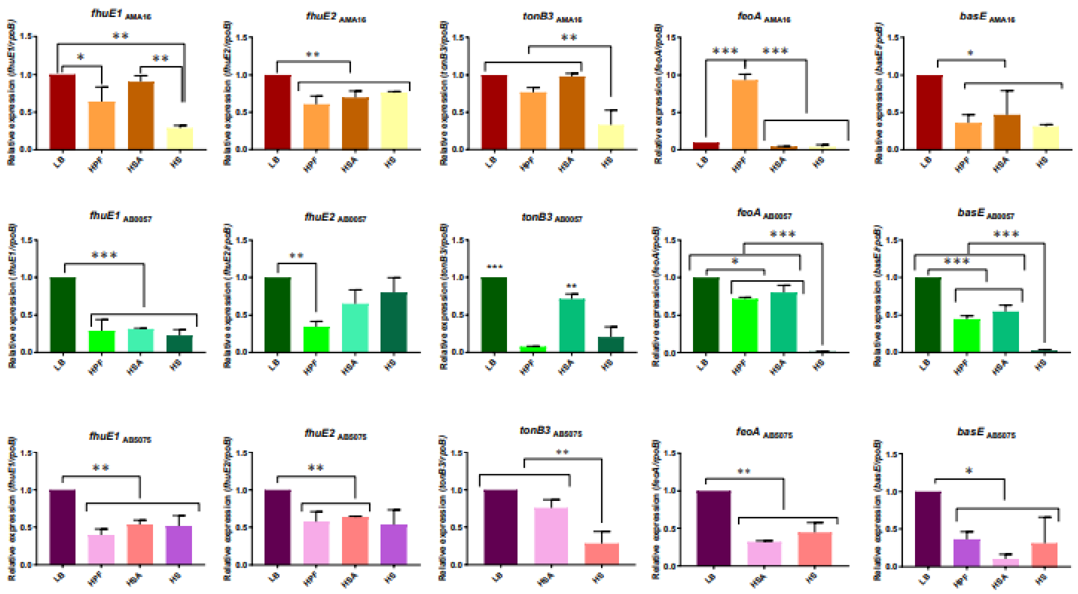

**Figure S1.** A) Heatmap outlining the differential expression of genes, *fhuE1*, *fhuE2*, *pirA* and *piuA* associated with iron uptake in presence of 4 % HPF or 0.2 % HSA. The asterisks represent the DEGs (adjusted p-value < 0.05 with. log2fold change >1). B) Differential expression of genes associated with iron-uptake obtained for *A. baumannii* AMA16, AB0057 and AB5075 strains cultured in the presence of HPF, HSA or HS.

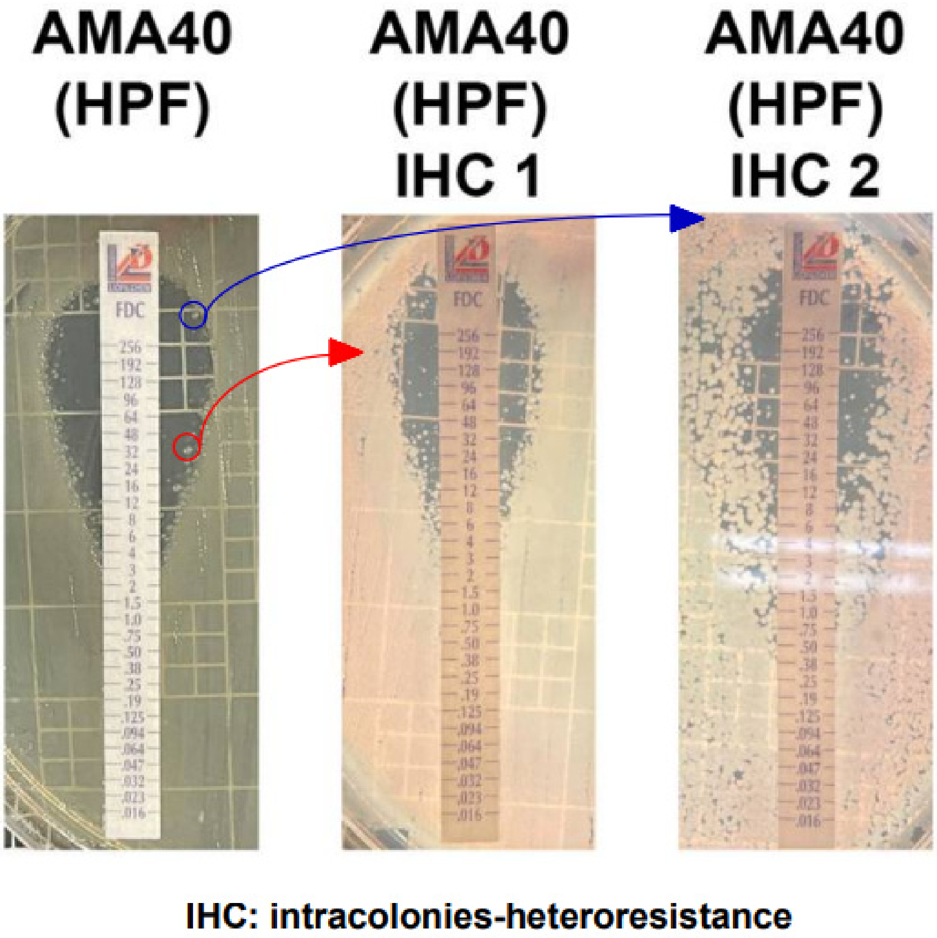


**Figure S2.** AMA40 CFDC MICs displaying colonies within the E-test zone and the MICs results of the selected intra colonies (IHC1 and IHC2). CFDC MICs were performed following the CLSI guidelines using CAMHA. The intracolonies obtained when AMA40 was exposed to 4% HPF were subculture and the CFDC MIC was performed.

**Table S1.** Comparison of the level of expression of iron associated and β-lactam resistance genes obtained by qRT-PCR in the three CRAB strains.

| Gene name | AMA16 ($Log_2FC$) | | | AB0057 ($Log_2FC$) | | | AB5075 ($Log_2FC$) | | |
|---|---|---|---|---|---|---|---|---|---|
| | HPF | HSA | HS | HPF | HSA | HS | HPF | HSA | HS |
| Iron Uptake | | | | | | | | | |
| *bauA* | -0.83 | -2.26 | -2.58 | -0.52 | -0.44 | -1.46 | -2.40 (1) | -0.81 | -1.37 |
| *basE* | -1.46 | -1.09 | -1.67 | -1.16 | -0.87 | -5.24 | -1.46 | -3.29 | -1.69 |
| *tonB3* | -0.38 | -0.03 | -1.58 | -3.71 | -0.49 | -2.29 | -1.95 (1) | -0,39 | -1.82 |
| *feoA* | 3.23 | -1.17 | -1.37 | -0.48 | -0.32 | -6.01 | -2.47 (1) | -1.64 | -1.17 |
| *pirA* | -1.23 | -0.21 | -0.90 | -2,04 | -1,32 | -0,44 | -1.71 | -1.06 | -0.37 |
| *piuA* | -1.58 | -0.20 | -1.34 | -1,05 | -0,59 | -1,51 | -0.19 | -1.01 | -1.76 |
| *fhuE1* | -0.64 | -0.14 | -1.77 | -1,80 | -1,68 | -2,15 | -1.34 | -0.90 | -0.96 |
| *fhuE2* | -0.72 | -0.52 | -0.40 | -1.52 | -0.62 | -0.32 | -0.80 | -0.66 | -0.90 |
| β-lactam resistance | | | | | | | | | |
| *pbp3* | 0.84 | 0.47 (2) | 2.46 (2) | 1.88 | 1.81 (2) | 2.67 (2) | 1.52 | 1.81 (2) | -0.41 (2) |

| | | | | | | | | | |
|---|---|---|---|---|---|---|---|---|---|
| *pbp1* | 0.72 | 0.12 (2) | 1.86 (2) | 1.35 | 0.89 (2) | 1.24 (2) | 2.10 | 0.89 (2) | 1.24 (2) |
| *carO* | 0.01 | -1.00 (3) | -1.39 | 0.71 | -0.86 (3) | 0.63 | -4.29 | -0.74 (3) | -0.74 (3) |
| *bla*$_{NDM-1}$ | -2.84 | -1.15 (3) | -1.62 | ND | ND | ND | ND | ND | ND |
| IS*Aba*125 | 0.88 | ND | 1.85 | ND | ND | ND | ND | ND | ND |
| *bla*$_{PER-7}$ | 0.84 | 0.96 | 1.33 (2) | ND | ND | ND | ND | ND | ND |
| *bla*$_{OXA-23}$ | ND | ND | ND | 1.21 | 0.77 (3) | 0.48 | 0.12 | 4.89 (3) | 4.81 (3) |
| *bla*$_{OXA-51-like}$ | ND | 4.50 (3) | ND | 0.88 | 4.00 (3) | 1.10 | 1.28 | 1.81 (3) | 3.25 (3) |
| *bla*$_{ADC}$ | ND | ND | ND | 0.16 | ND | 0.67 | 2.48 | ND | 2.68 |
| *bla*$_{GES-14}$ | ND | ND | ND | ND | ND | ND | -6.64 | ND | -3.01 |

(1) Pimentel *et al*, 2021,A ; (2) Pimentel *et al*, 2021,B; (3) Le *et al*, 2021

Log$_2$FC > 1 ($p<0.05$)    og$_2$FC < - 1 ($p<0.05$)

Log$_2$FC (0-1)    Log$_2$FC (-1-0)

ND: not determined
